# Stellate ganglion block using procaine in the treatment of herpes zoster ophthalmicus with keratitis after root canal treatment and post-herpetic pruritus in the third trimester of pregnancy and early lactation: a case report and review of the literature

**DOI:** 10.3389/fmed.2026.1830835

**Published:** 2026-05-07

**Authors:** Zoltan Kovacs, Borislava Spasova, Henrik Szoke

**Affiliations:** 1 Department of Obstetrics, Triton Robert Hospital, Budapest, Hungary; 2 Kantonspital Baselland Gesundheitszentrum Laufen, Laufen, Switzerland; 3 Faculty of Health Sciences, Doctoral School of Health Sciences, University of Pécs, Pécs, Hungary; 4 Department of Integrative Medicine, Faculty of Health Sciences, University of Pécs, Pécs, Hungary

**Keywords:** case report, herpes zoster ophthalmicus, neural therapy, pregnancy, procaine, root canal treatment, stellate ganglion block

## Abstract

**Introduction:**

Herpes zoster results from the reactivation of the varicella zoster virus, which rarely occurs during pregnancy. This condition increases maternal morbidity, and the main complications are herpes zoster ophthalmicus and post-herpetic neuralgia. This report presents the first known case of herpes zoster ophthalmicus with keratitis after a root canal procedure and post-herpetic pruritus in the third trimester of pregnancy and early lactation. Neural therapy was used as a complementary treatment, known for its analgesic and anti-inflammatory effects.

**Case presentation:**

A 41-year-old multigravida in the 28th gestational week, without prodromal symptoms, developed a characteristic herpes zoster ophthalmicus rash 1 day after undergoing an upper-right 7-tooth root canal. Within the first 3 days of the first blister, the patient was hospitalized and administered intravenous and topical acyclovir as a state-of-the-art treatment to alleviate symptoms and help the keratitis subside. After hospitalization, the patient experienced severe acute herpes zoster pain accompanied by itching and numbness. In addition to oral and topical acyclovir and paracetamol therapy, intra- and subcutaneous neural therapeutic injections of 1% procaine in the temporal and supraorbital regions and a stellate ganglion block were administered as potential treatment methods to improve quality of life. The decrease in pain and itching was measured using the Numeric Rating Scale. After 4 weeks, severe pain ceased, and after 8 weeks, severe itching disappeared during the breastfeeding period. Neither pain nor itching recurred during the 47-month-long follow-up.

**Conclusion:**

Although the self-limiting course of shingles has minimal effects on the fetus, maternal complications can cause a significant burden. Data on the treatment of herpes zoster ophthalmicus during pregnancy and lactation are limited. Acyclovir and paracetamol—as important pillars of the treatment—were supplemented with neural therapy, which included stellate ganglion blocks. Due to the limitations of the case report, neural therapy should be confirmed in large clinical trials as a complementary method for herpes zoster ophthalmicus during pregnancy.

## 1 Introduction

A systematic review of 69 articles on the prevalence of herpes zoster (HZ) in the general population found that the prevalence rate was higher in women than in men (with rates of 6.05–12.8 per 1,000 individuals for women compared to 4.3–8.5 per 1,000 individuals for men) (1). This difference is likely due to a non-specific immune response to latent viral infections (2). Pregnancy is associated with changes in maternal hormonal and immune conditions (3), leading to relative immunosuppression (4). Although no specific studies are available, the incidence of HZ in pregnant women is estimated to be 1 in 20,000 pregnancies (4). According to a 2020 meta-analysis of risk factors for HZ infection, immunodeficiency, family history of HZ, malignancy, older age, and physical trauma can increase the risk of varicella zoster virus (VZV) reactivation (5); it can also be triggered by dental treatments that irritate the trigeminal nerve (6). HZ (shingles) and its complications can significantly impact maternal health, but they do not normally increase fetal mortality or lead to congenital malformations (7). After primary infection, VZV stays dormant in the ganglia of the cranial and spinal nerves and may reactivate later in life as HZ (8). Clinically, HZ is diagnosed by the typical appearance of a unilateral dermatomal distribution of clustered vesicular blisters, often preceded by a prodromal phase of pain and segmental discomfort, characteristic of zoster. There are three phases of HZ reactivation: acute HZ-related pain, subacute HZ-related pain, and post-herpetic neuralgia (PHN). Acute HZ-related pain lasts up to 30 days. Subacute herpes neuralgia is a painful condition that persists after the blisters have healed but resolves within 3 months of onset. There is no agreement on the definition of PHN, but it is generally defined as pain that persists for more than 3 months after the onset of the disease (9). Close observation is important to reduce the risk of complications such as PHN and herpes zoster ophthalmicus (HZO), which result from VZV reactivation in the ophthalmic segment of the trigeminal nerve (V/1) (10, 11). Post-herpetic pruritus (PHP) is relatively underreported and is a frequently neglected accompanying component of PHN (12). HZO is associated with a higher risk of PHN than HZ in any other location (13).

Some studies have confirmed the anti-inflammatory effect of local, anesthetics (LAs) and their combined use, which includes local (supra) segmental treatment (14–16). Sympathetic and somatic nerve blocks using LAs and/or corticosteroids have also been used to control pain during the acute phase of HZ and PHN, as well as to reduce the incidence of PHN (17, 18). LAs include lidocaine and procaine. Using therapeutic local anesthesia—known as neural therapy—for the treatment of pain, inflammation, and other medical conditions is well attested in integrative medicine (19–22). This is the first article that describes a case of acute HZO with keratitis after root canal treatment and PHP during the third trimester of pregnancy and early lactation, which was treated with acyclovir, paracetamol, and neural therapy.

## 2 Case presentation

### 2.1 Patient’s medical history

Surgery: The patient had one previous cesarean section, one spontaneous miscarriage, and a negative Pap smear in 2022. There were no records of drug sensitivity, but she did have a history of chickenpox infection in childhood and no known high risk for PHN. Diseases: The patient had Hashimoto’s hypothyroidism and was taking 125 mcg of thyroxine daily. During her pregnancy, she experienced a weight gain of 11 kg, with a BMI of 27.68 at the start of her HZO. Until the onset of HZ, the pregnancy was without any complications.

### 2.2 Clinical findings

**Figure 1:**
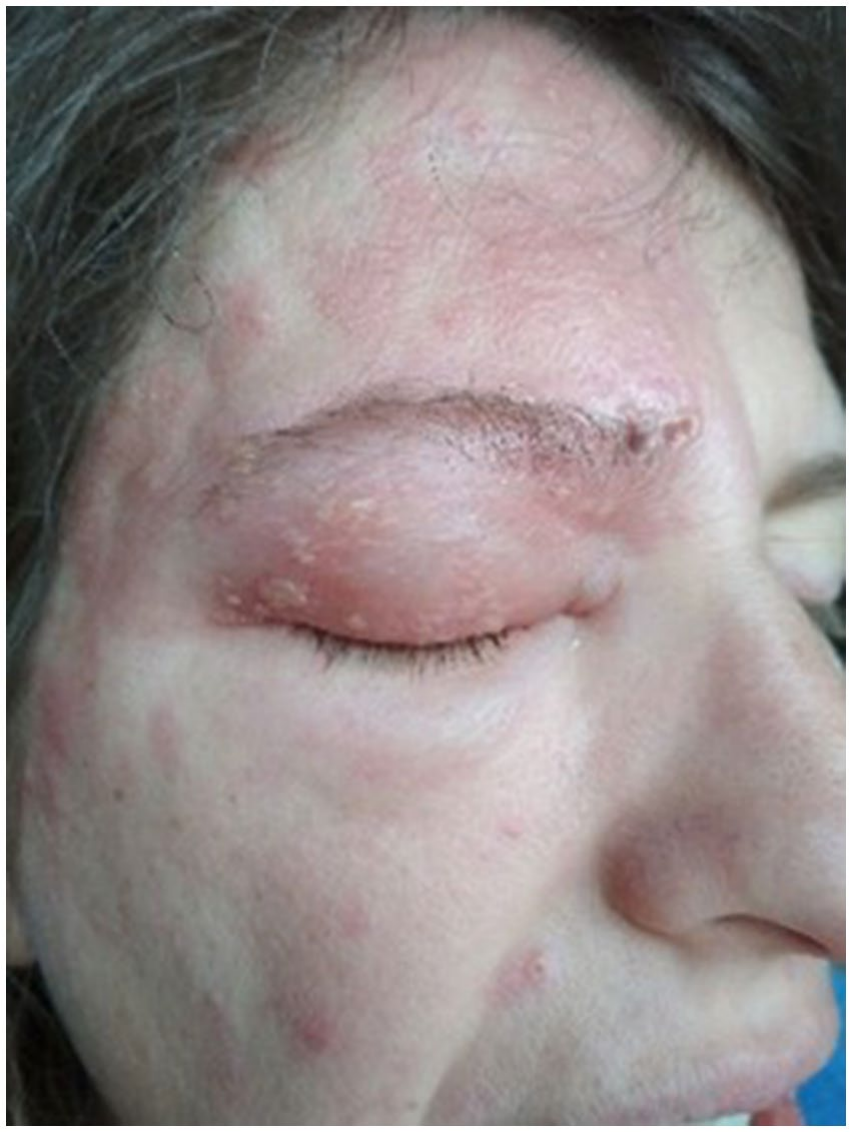
First day of hospitalization.

Upon admission to the hospital on 6 February, the rash spread further on the face of the 41-year-old patient who was 28 weeks pregnant (Figure 1). Routine laboratory tests showed elevated levels of CRP and neutrophil granulocyte counts. The patient also experienced an increased pulling and burning sensation in the affected regions. A COVID-19 test returned negative results. A pressure-sensitive lymph node, approximately 5 mm in size, was palpable behind the right ear. There were no signs of fever or cold shivers.

The timeline of symptoms and medical care is shown in Table 1.

**Table 1:** Timeline of symptoms and medical care.

Dates	Summaries of initial and follow-up visits	Diagnostics	Treatments
3 February 2022	A throbbing toothache at night	–	–
4 February 2022	First blister under the right eyebrow	Dental examination	A root canal on the upper right 7th tooth
5 February 2022	narrowing of the right eyelid opening, and an inflamed right upper eyelid, with clusters of 1–4 mm serous vesicles	Dermatology; ophthalmology: herpes zoster ophthalmicus	5 × 800 mg acyclovir daily; octenidine skin disinfectant
6 February 2022	Increasing number of blisters; eye opening was not possible	Start of hospitalization; elevated C-reactive protein and neutrophil granulocyte counts	3 × 500 mg i.v. acyclovir daily; octenidine; oral acyclovir discontinued
7 February 2022	More blisters in the supraorbital region	No signs of zoster in the oral mucosa	A root canal on the upper right 7th tooth; insertion of a temporary filling
8 February 2022	The pregnant woman could not open her right eye; periocular edema	Herpes zoster ophthalmicus with keratitis in the right eye	Acyclovir gel; preservative-free artificial tears, five times daily; octenidine; i.v. acyclovir
10 February 2022	For the first time, she could see the sign on the vision test with her right eye	Keratitis and blisters seemed to decrease	Acyclovir gel; preservative-free artificial tears; octenidine; i.v. acyclovir
11 February 2022	Discharging from the hospital	The keratitis subsided. No abnormalities in the baby’s development	i.v. acyclovir discontinued; 5 × 800 mg acyclovir tablets daily; ongoing therapy^1^
20 February 2022	Severe pain; itching and numbness appeared in the right supraorbital; most of the scabs disappeared	Dermatology	Ongoing therapy^2^; 4 grams of paracetamol per os daily
22 February 2022	Complaints of headache; facial ache in the right supraorbital region	Dermatology	3x vitamin B capsules a day orally; acyclovir tablets discontinued after 11 days of intake; ongoing therapy^3^
24 February 2022	Severe pain; itching and numbness appeared in the right supraorbital and temporal region	Physical examination in NT	Ongoing therapy^4^; first NT; paracetamol discontinued
1 March 2022	Photosensitivity, eye pain, and narrowing of the right eyelid opening	Ophthalmology: no more keratitis	Vitamin B; acyclovir gel discontinued; ongoing therapy^5^
24 March 2022	No more serious pain returned, but severe pruritus started in the supraorbital region	–	Ongoing therapy^5^
19 April 2022	–	–	C-section
20 July 2022	No more severe itching	Physical examination in NT	NT
7 December 2022	No more severe numbness could be detected	Physical examination in NT	NT

i.v., intravenous; NT, neural therapy. ^1^Octenidine skin disinfectant, acyclovir topical gel, preservative-free artificial tears. ^2^Octenidine skin disinfectant, acyclovir topical gel, preservative-free artificial tears, 5 × 800 mg acyclovir tablets daily. ^3^Octenidine skin disinfectant, acyclovir topical gel, preservative-free artificial tears, 5 × 800 mg acyclovir tablets daily, paracetamol for pain relief. ^4^Octenidine skin disinfectant, acyclovir topical gel, preservative-free artificial tears, paracetamol for pain relief, vitamin B capsules 3 times a day orally. ^5^Octenidine skin disinfectant, preservative-free artificial tears, neural therapy, procaine eye drops.

### 2.3 Assessment of pain and pruritus intensity

Pain and pruritus intensities were assessed using the numeric rating scale (NRS) (23, 24). The report of this case was based on the CARE guidelines (25).

### 2.4 Course of herpes zoster ophthalmicus

At the appointment in the first author’s office following her discharge, the gravida agreed to undergo neural therapy as a personal preference because of her severe pain.

**Figure 2:**
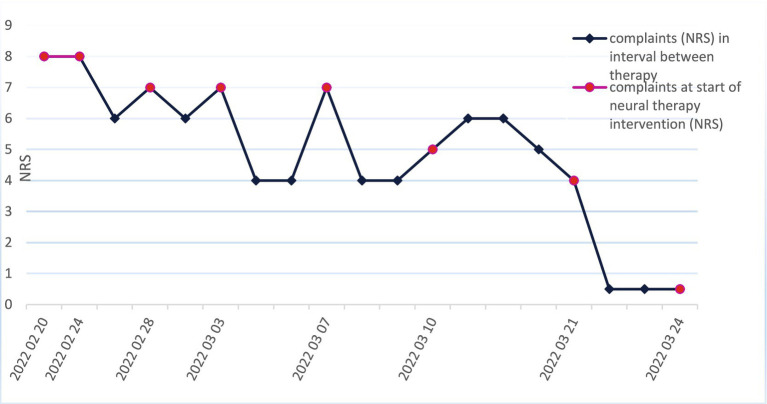
Complaints of pain and the course of neural therapy Y-scale: NRS (0–10). Red oval dots: Complaints (NRS) at the beginning of neural therapy intervention Rhomboid dots: Complaints (NRS) in the time interval between therapy appointments, according to the patient’s estimation of the average NRS during the last weeks. NRS 0–2 remained constant without intermediate deterioration since 24 March 2022.

**Figure 3:**
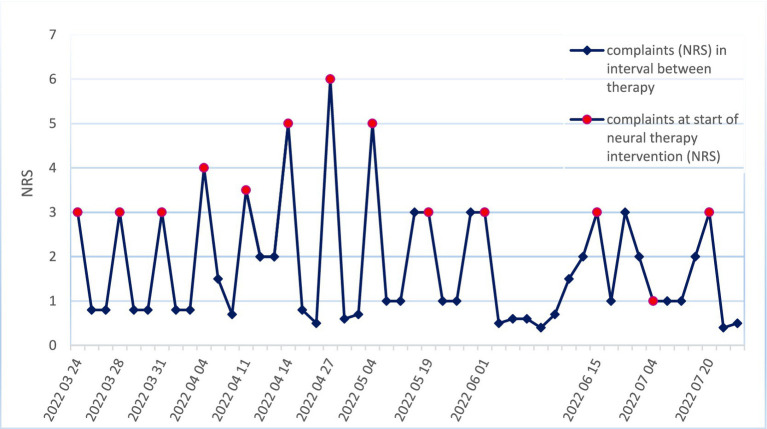
Complaints of pruritus and the course of neural therapy Y-scale: NRS (0–10). Red oval dots: Complaints (NRS) at the beginning of neural therapy intervention. Rhomboid dots: Complaints (NRS) in the time interval between neural therapy appointments, according to the patient’s estimation of the average NRS during the last weeks. NRS 0–2 remained constant without intermediate deterioration since 20 July 2022.

After six treatments, significant pain reduction was observed (Figure 2). After 13 additional treatments, a considerable decrease in itching was observed (Figure 3). The disappearance of numbness (sensory disturbance) in the supraorbital and temporal areas was observed 9.5 months (07 December 2022) after the onset of neural therapy.

## 3 Discussion

The well-known self-limiting nature of shingles could complicate our case. According to one study, shingles typically resolves spontaneously within 3 months; 18.8% of patients continue to experience pain, and after 12 months, this proportion decreases to 7.6% (26). In approximately 15% of HZ patients, PHN lasts longer than 3 months (27). HZO accounts for approximately 10–20% of HZ cases, and the lifetime risk of developing it is approximately 1% (28). With regard to the possibility of spontaneous recovery of HZ and, to a lesser extent, PHN (9), as in our case study. It took the gravida almost 4 months to complete, covering the second half of the pregnancy and the early lactation period. In a multicenter retrospective study, involving the outcomes of 72 pregnant women between 2010 and 2024, no adverse maternal or fetal outcomes of HZ itself or its antiviral treatment were observed, although 51.4% of the gravidas remained without any drug treatment (29). A Mexican study involving 17 pregnant women suggested that HZ during pregnancy typically has a benign course and does not lead to maternal or fetal complications (30). It is difficult to predict who will not recover or how long the pain will last. Antiviral medications are included in previous treatment guidelines (23) and play a key role in HZO treatment. Acyclovir may be an additional complicating factor in this case. Acyclovir accelerates the disappearance of blisters and the healing of skin lesions and prevents the development of new lesions by inhibiting VZV replication and further cell infection, likely reducing nerve damage. This controls the infection and helps the immune system fight it. Therefore, treatment should be initiated as soon as possible. High-quality evidence indicates that oral acyclovir does not significantly reduce the incidence of PHN (31). The risks of maternal HZ during pregnancy remain undefined (32). In our case, the pregnant woman developed a complicated form of HZO characterized by severe pain, itching, and numbness, which significantly impaired her quality of life. In addition to oral and topical acyclovir and paracetamol as early intervention—for ethical reasons and considering the patient’s individual needs and preferences—we initiated inexpensive, easily performed outpatient neural therapy, including SGB, rather than waiting for the HZ to resolve spontaneously in the third trimester of pregnancy. Antiepileptic drugs play a significant role in the treatment of pain associated with acute HZ and PHN (9). However, almost all systemic adjuvant drugs currently in use (e.g., gabapentin and TCAs) have common dose-limiting side effects: drowsiness, reduced cognitive concentration, and nausea, and TCAs also have anticholinergic side effects (mainly sedation and dry mouth) (33). These side effects should be avoided during pregnancy. In a scoping review with meta-analysis, the use of gabapentin was associated with an increased risk of admission to the neonatal intensive care unit (NICU) (34). A population-based cohort study concluded that maternal gabapentin use, particularly in the late stages of pregnancy, is associated with an increased risk of preterm birth, low birth weight for gestational age, and admission to the NICU (35). The most commonly recommended medications for relieving severe pain during pregnancy are paracetamol, non-steroidal anti-inflammatory drugs (NSAIDs), and opioids [31]. According to reports on the use of acetaminophen to treat severe pain associated with HZ neuralgia in pregnant women, the newborns were healthy, as in our study (13, 36, 37). NSAIDs are generally used to treat mild-to-moderate pain and fever. After 30 weeks of pregnancy, NSAIDs are contraindicated because they can cause premature closure of the fetal ductus arteriosus. Opioids such as codeine and oxycodone are used to treat moderate-to-severe pain. The main concern with these drugs is that prolonged use can lead to dependence in the mother, which can lead to withdrawal symptoms in the newborn; thus, they should be avoided during pregnancy (33). Patients with HZO are at an increased risk of developing corneal ulcers and should be carefully examined and counseled (38).

The inflammation was eliminated locally with a root canal. On the one hand, this was important because the dentin and pulp are connected to the nervous system via the somatic trigeminal nerve, including nociceptive neurons and afferent sympathetic fibers. The supply of sympathetic fibers to the dentin and the pulp connects the teeth with the autonomic nervous system (ANS), including SG (39). On the other hand, the necessary root canal treatment might be a trigger, due to its irritation of the trigeminal nerve, enhancing the reactivation of VZV (6), but this is not sufficiently supported by the literature. From a neuroimmunological perspective, the pathological neuroimmune communication can be efficiently modulated by LAs—in particular, through interventions such as SGBs that can “revert” these dysregulated neuronal tracks (16)—since Fischer postulated a singular pathogenetic process in the neuroimmune system, mainly under the control of the ANS (16). The Heidelberg University Neural Therapy Education & Research Group regularly publishes new research (40, 41) and organizes an annual international hybrid conference on evidence-based neural therapy. A review article on complementary therapies included neural therapy as early as 2011 (42). A national survey showed that neural therapy was among the most commonly used complementary treatments by general practitioners in Germany (43). Neural therapy has also been documented as a neural therapeutic medicine (44). A new membrane theory of local anesthetics explains the diversity of their molecular effects in addition to sodium channel blockade (15, 41).

Local procaine injections into the right (supra) orbital, frontal, and temporal segments may inhibit the peripheral sensitivity of the nociceptive neurons (14) and reduce sympathetic afferent coupling, while improving microcirculation (45). Hence, they may counteract peripheral sensitivity.

Procaine eyedrops may enhance wound healing in the cornea, due to their pain-relieving and anti-inflammatory effects (46). In our case, we used SGB with procaine to treat acute HZO pain in pregnancy and complex unilateral somatic and sympathetic blocks in the same therapeutic setting many times. The pain-relieving effect of neural therapy might have improved significantly when SGB injections were added to the treatment from the third session onwards. We used the injection method for SGB, as described by Puente and Fischer (47). Furthermore, a systematic scoping review and a double-blind, placebo-controlled study found no significant adverse effects of procaine (48, 49), and it does not interact with other drugs because it is degraded by pseudocholinesterase, which is present everywhere in the body (16). All the injections were performed by the first author, and 10–15 mg of 1% procaine was used each time. According to the e-lactancia information website (50) procaine is fairly safe during breastfeeding when administered topically (e.g., eyedrops), locally intracutaneously, subcutaneously, or intramuscularly, or as a nerve block injection. Excretion in breastmilk is very unlikely.

There were no serious adverse effects of the 1% procaine injections, with transient Horner’s triad (because of the successful SGB). These injections were generally well-tolerated. In some cases, mild dizziness and orthostatic dysregulation occurred, lasting 5–7 min. The patient was informed about the correct use of LA eyedrops containing procaine at home to avoid accidental eye injuries.

A 2025 evidence-based systematic review of HZ and PHN (9) described that the most important treatment for acute HZ-related pain is antiviral therapy within 72 h of symptom onset. This was performed in our patient’s case. There is currently no consensus on the most effective treatment for PHN (51). Additional symptomatic treatment options include analgesics in accordance with the World Health Organization pain management ladder, tricyclic antidepressants (e.g., nortriptyline), and anticonvulsants (e.g., gabapentin). If pain does not decrease sufficiently, interventional treatments, such as epidural injections with local anesthetics and corticosteroids or pulsed radiofrequency treatment of the dorsal root ganglion, may be considered. Transdermal capsaicin, lidocaine, or oral medications such as antidepressants or antiepileptics are preferred for the treatment of PHN. The study also reported low-quality evidence regarding the use of sympathetic blocks (SGBs) for the treatment of pain associated with acute HZ, but there is no evidence for their use in the treatment of PHN (9).

A 2017 Korean systematic review and meta-analysis described (52) that in patients with acute HZ, the use of nerve blocks during the acute phase of the disease to prevent PHN shortens the duration of zoster-related pain, and the use of somatic blocks (including paravertebral and repeated/continuous epidural blocks) is recommended for the prevention of PHN. SGBs do not reduce the incidence of postherpetic neuralgia, but multiple blocks may have beneficial effects. All studies reported varying rates of PHN incidence, defined as persistent pain and/or abnormal sensation in dermatomes affected by HZ. In our patient, abnormal sensations, including numbness, persisted for several months following the onset of HZO.

According to a systematic review and network meta-analysis published in 2021 on pharmacological and non-pharmacological strategies for the prevention of PHN (53), continuous epidural blockade with LAs and steroids, antiviral agents combined with intradermal or subcutaneous injections of LAs and steroids, and paravertebral blocks combined with antiviral and antiepileptic agents are effective in preventing PHN.

The evidence-based, multidisciplinary, *de novo* clinical guidelines developed by the Korean Pain Society for the treatment of refractory PHN (54) addressed the use of opioids and analgesic interventions, such as nerve blocks, in patients who do not respond to first-line pharmacotherapy. It defined PHN as postherpetic pain persisting for more than 1 month after the onset of the rash. The guideline found very low levels of evidence for the use of tramadol or the tramadol/acetaminophen combination, strong opioids, epidural blocks for pain relief, nerve blocks, and SGB in patients with refractory PHN affecting the facial area, a low level of evidence for type A botulinum toxin injections and pulsed radiofrequency therapy, and a moderate level of evidence for spinal cord stimulation.

However, there is a relative paucity of research regarding the treatment and prognosis of HZ in gravidas (29). Due to the rarity of neural therapy with procaine, HZO, and PHP in pregnancy and lactation, evidence from randomized controlled trials is lacking; therefore, this case provides insight into this unique condition.

PHP can occur in conjunction with PHN or on its own, demonstrating a partially independent mechanism. This neuropathic itching does not respond to antihistamines or NSAIDs, which are effective for normal itching. Furthermore, not all drugs that are effective for PHN relieve PHP. In fact, opioids—which are the common treatment for severe pain relief—can cause itching. An LA injection into the itchy area often provides immediate and temporary relief, demonstrating the importance of residual afferent signals in modulating PHP, although it does not rule out a central role in its pathophysiology. One objective of treating acute HZO and PHP is to improve quality of life, as the disease can negatively affect physical and emotional functioning and sleep (26, 55). A 47-month follow-up showed no vision problems or PHN.

### 3.1 Review of the literature

To the best of our knowledge, two case reports have discussed pregnancies complicated by spontaneously occurring trigeminal HZ. However, unlike our case report, none of these publications were based on the CARE guidelines. There was no long-term follow-up (e.g., 6–47 months) to monitor PHN or PHP after HZO, nor was there a description of analgesic therapy, in contrast to our case. In the 2007 African case, a 7-month pregnant woman who was human immunodeficiency virus**-**positive had maxillary HZ with corneal involvement. The keratitis (corneal involvement) resolved with antiretroviral therapy and systemic and topical acyclovir treatment (56). In 2014, an Indian case report described a 21.5-week pregnant patient. This case was considered rare because two branches of the trigeminal nerve were simultaneously affected in the form of HZO without keratitis and PHN, along with orocutaneous lesions of the maxillary zoster in an immunocompetent nulliparous pregnant woman (57). No therapeutic intervention was mentioned in this article.

This case highlights the need to consider treatment strategies for severe pain and pruritus during pregnancy, especially at and later than the 30th gestational week, when NSAIDs are no longer safe, and paracetamol may not be helpful.

### 3.2 Strength and limitations

SGB performed correctly with procaine injections (therapeutic local anesthesia) is a low-risk, cheap treatment option (58). However, this therapeutic method has certain limitations. In most cases, multiple treatments are necessary.

The merits of this case report are as follows: its novelty, the generation of a hypothesis for subsequent research, the narrative aspect of a rare case, and its low cost. This article also has educational value for both healthcare providers and patients. The limitations of this case report are as follows: its descriptive and non-controlled nature; the lack of control for confounding factors (such as acyclovir therapy and the self-limiting nature of HZ); the lack of generalizability; the lack of a cause–effect relationship; and the potential for overinterpretation, selection, hindsight, and recall bias (59). Case reports are at the bottom of the evidence hierarchy.

## 4 Conclusion

We reported the first case of acute HZO developing in the third trimester of pregnancy following root canal treatment, as well as its association with keratitis and PHP in the third trimester of pregnancy and lactation. Research and data on the treatment of HZO during pregnancy and lactation are limited. In addition to drug therapy with acyclovir and paracetamol, which are integral parts of HZO treatment guidelines, it is hypothesized that neural therapy using SGB, which has a low level of evidence, might help pregnant women alleviate symptoms, considering the self-limiting nature of HZO. Further rigorous studies are needed to confirm the efficacy of neural therapy for HZO during pregnancy as a complementary option to conventional treatments.

## Data Availability

The original contributions presented in the study are included in the article/supplementary material, further inquiries can be directed to the corresponding author.
